# Exercise-induced intra-ventricular gradients as a frequent potential cause of myocardial ischemia in cardiac syndrome X patients

**DOI:** 10.1186/1476-7120-6-3

**Published:** 2008-01-14

**Authors:** Carlos Cotrim, Ana G Almeida, Manuel Carrageta

**Affiliations:** 1 Cardiology Department, Garcia de Orta Hospital, Almada, Portugal; 2 Cardiology Department, Santa Maria Hospital, Lisboa, Portugal; 3 Lisboa University Medical School, Lisboa, Portugal

## Abstract

**Background:**

The development of intra-ventricular gradients (IVG) during dobutamine or exercise stress is not infrequent, and can be associated to symptoms during stress.

The purpose of this study was to assess the occurrence of IVG during exercise stress echocardiography in cardiac syndrome X patients.

**Methods:**

We prospectively evaluated 91 patients (pts) mean aged 51 ± 12 years (age ranged 20 to 75 years old), 44 of whom were women. All pts had angina, positive exercise ECG treadmill testing, normal rest echocardiogram and no coronary artery disease on coronary angiogram (cardiac X syndrome). After complete Doppler echocardiographic evaluation with determination of left ventricular outflow tract index (LVOTi), relative left ventricular wall thickness (RLVWT) and left ventricular end-diastolic volume index (LVDVi), all patients underwent stress echocardiography with two-dimensional and Doppler echographic evaluation during and after treadmill exercise.

**Results:**

For analysis purpose patients were divided in 2 groups, according to the development of IVG. Doppler evidence of IVG was found in 33 (36%) of the patients (Group A), with mean age 47 ± 14 years old (age ranged 20 to 72 years) and with a mean end-systolic peak gradient of 86 ± 34 mmHg (ranging from 30 to 165 mmHg). The IVG development was accompanied by SAM of the mitral valve in 23 pts. Three of these pts experienced symptomatic hypotension. Ten were women (30% pts). 58 pts in group B, 34 of whom were women (59%) (p = 0,01 vs group A), mean aged 53,5 ± 10,9 years old (age ranged 34 to 75 years) (p = 0,03 vs group A), did not develop IVG. LVOTi was 10,29 ± 0,9 mm/m^2 ^in group A and 11,4 ± 1 mm/m^2 ^in group B (p < 0,000); RLVWT was 0,36 ± 0,068 in group A and 0,33 ± 0,046 in group B (p < 0,01); LVDVi was 44,8 ± 10 ml/m^2 ^in group A and 56 ± 11,6 ml/m^2 ^in group B (p = 0,000).

**Conclusion:**

1. A significant number of patients with cardiac X syndrome developed IVG during upright exercise in treadmill. These pts (group A) are mainly males and younger than those who did not develop IVG.

2. The development of IVG and mitral valve SAM on exertion seems to be associated with ST segment downsloping during stress testing in patients without epicardial coronary disease.

3. The development of IVG and mitral valve SAM seems to be associated with lower LVOTi, lower LVDVi and higher RLVWT.

## Background

The development of IVG during DSE has been largely reported and this fact is commonly associated with symptoms during the stress study [1,2]. The occurrence of IVG during the ESE is rarely find [3]. In a group of 10 patients who developed IVG during DSE, we performed ESE and we found a small IVG in only one of them [4]. In a 23 years old male, with a positive treadmill test, a structural normal heart, normal coronary angiographies, an ESE was performed and during the study we unexpectedly detect a 102 mmHg intra-ventricular gradient [5] and systolic anterior movement of mitral valve (SAM). A similar case has been reported previously by Lau [6] and was treated successfully with β blockers.

The aim of this study was to present the results of search for intra-ventricular gradients during exercise stress echocardiography in patients with angina, positive stress electrocardiography, normal coronary arteries, and normal echocardiogram (cardiac X syndrome).

## Methods

This study includes 91 (pts) mean aged 51 ± 12 years (age ranged 20 to 75 years old), 44 of whom were women. All pts had angina, positive exercise ECG treadmill testing (four patients had only ischemia in a myocardial perfusion study), normal rest echocardiogram – no left ventricular hypertrophy – and no coronary artery disease on coronary angiogram. Diabetes mellitus or uncontrolled hypertension in the last year were motives of exclusion.

Twenty four patients (26%) are current smokers and thirty three pts (36%) had hypercholesterolemia.

At the moment of inclusion in the study, 47 (51%) patients were treated with nitrates, 10 (11%) with calcium antagonists, 18 pts (20%) on β blockers, 12 pts (13%) with angiotensin II receptor blockers or angiotensin-converting enzyme inhibitors, 7 pts (8%) with diuretics.

All patients gave informed consent for the study.

### Exercise stress echocardiography

**Figure 1:**
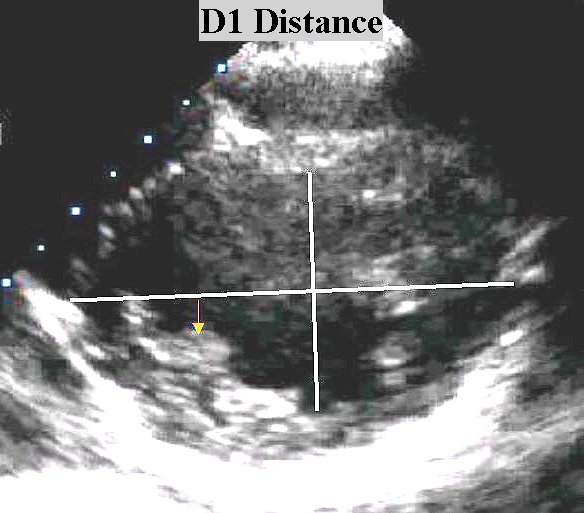
A line that originates at the point where the inferior wall begins, divides the left ventricle in halfs. The D1 distance is the distance between that line and the postero internal papillary muscle (arrow) at the point where it encounters the inferior wall.

**Figure 2:**
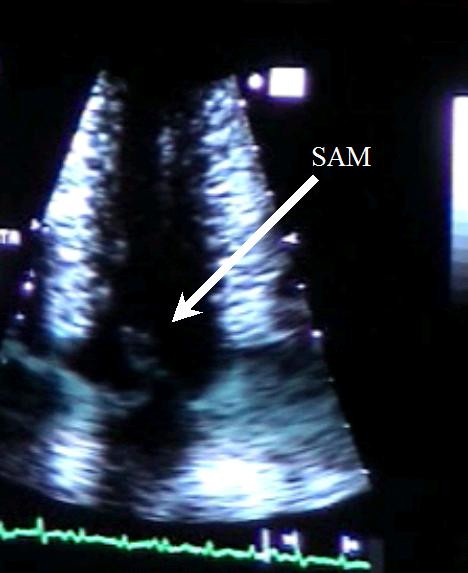
In this apical four chamber view obtained near peak exercise (before stoping) we can clearly see systolic anterior movement of mitral valve.

**Figure 3:**
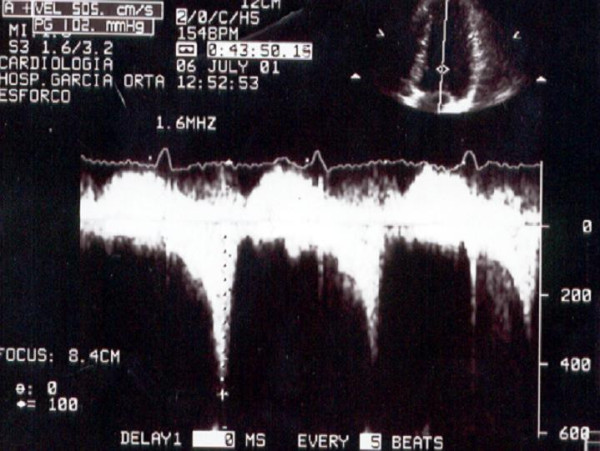
The flow obtained at that moment with continuous Doppler.

After complete echocardiographic evaluation which also includes determination of left ventricular outflow tract index (LVOTi), relative left ventricular wall thickness (RLVWT) and left ventricular end-diastolic volume index (LVDVi), all patients underwent stress echocardiography with two-dimensional and Doppler echographic evaluation. We also measured the distance D1 in the end of diastole, in short axis view, as showed in Figure 1. Exercise stress echocardiography as performed by the authors [7] includes evaluation during all the exercise in treadmill, of contractility, and in this group of patients also pulsed, continuous and colour Doppler from apical window (Additional Files 1 and 2). Mitral valve motion was also assessed, for the development of SAM (Additional file 3 and Figure 2). The exam was totally stored in videotape and partially in optical disk. A significant intraventricular gradient, was considered an increase in the intraventricular flow velocity to or greater than 2.5 m/s at the end of systole (telesystolic peak)(Figure 3) and its occurrence separated the patients in two groups.

### Statistical analysis

The results are expressed as mean ± SD for continuous variables, and frequency percentage for categorical variables. The variables were compared between groups with the *student *T test. The X^2 ^test was used for qualitative variables. Results of statistic tests were considered significant if the observed p value was less than 0.05.

## Results

**Figure 4:**
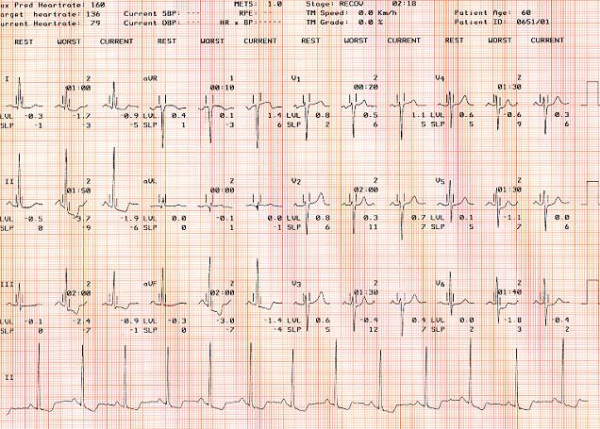
Summary of a positive exercise stress test in one patient from the study.

**Figure 5:**
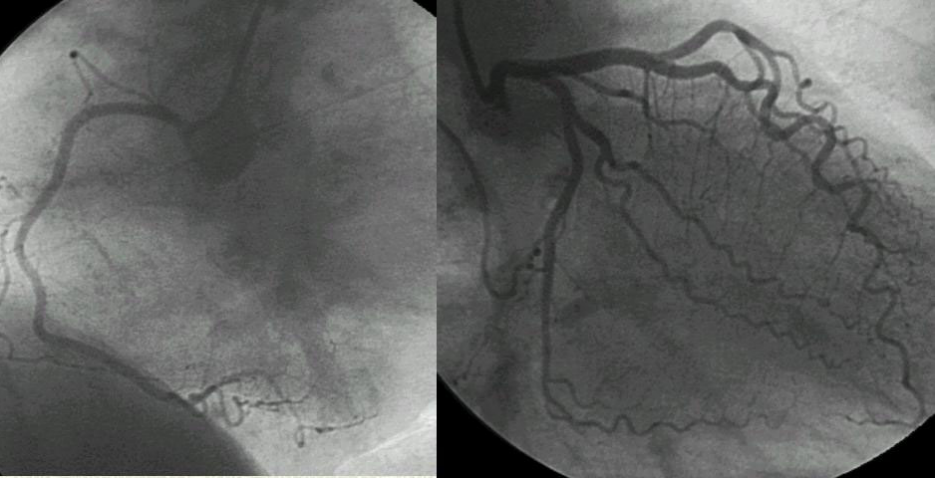
Normal angiography of coronary arteries in the same patient.

A typical example of stress electrocardiography (Figure 4), and angiographic (Figure 5) findings is showed.

From the all group, 33 patients (36%) develop IVG (group A) and 58 pts (64%) did not develop intraventricular gradient (Group B) as defined by the authors. In group A the IVG at peak exercise was 86 ± 34 mmHg (ranging from 30 to 165 mmHg).

In all but 11 patients, 85% of predicted maximum theoretical heart rate for age was reached. Clinical and demographic data is presented in Table 1, details of the exercise test are depicted in Table 2, and details of echocardiogram in Table 3, 4, and 5.

**Table 1:** Clinical and demographic data

	Group A	Group B	p
Age, years	47,70 ± 13,36	53,53 ± 10,89	0,026
Sex, female (%)	10/33 (30%)	34/58 (59%)	0,008
BSA m^2^	1,8 ± 0,16	1,73 ± 0,13	0,022
Effort Angina	28/33 (85%)	33/58 (56%)	0,006
Effortless Angina	9/33 (27%)	35/58 (57%)	0,002
Duration of symptoms before cath. (months)	15 ± 10	46 ± 40	0,000
Time of FLW (months)	36,4 ± 17,9	39,1 ± 19,5	0,55
Events in FLW	6/33 (18%)	8/56 (14%)	0,31
ACS in FLW	1/33 (3%)	7/56 (13%)	0,24
β Bloq.	7/33 (21%)	11/58 (19%)	0,798
CCB	4/33 (12%)	7/58 (12%)	0,666
Nitrates	16/33 (48%)	31/58 (53%)	0,769
IECA/ARAII	5/33 (15%)	7/58 (12%)	0,680
Diuretics	2/33 (6%)	5/58 (9%)	0,663
β Bloq. FLW	20/33 (60%)	17/56 (30%)	0,003
CCB FLW	4/33(12%)	19/56 (34%)	0,530
Nitrates FLW	9/33 (27%)	33/56 (59%)	0,006
IECA/ARAII FLW	9/33 (27%)	8/56 (14%)	0,068
Diuretics FLW	4/33(12%)	4/56 (7%)	0,403

BSA – body surface area; ACS – Acute coronary Syndrome; CCB – Calcium chanell blockers; FLW – follow-up

**Table 2:** Exercise test data

	Group A	Group B	p
HR Baseline	70 ± 10,5	70 ± 11	0,769
HR Peak	163 ± 14	151 ± 17	0,001
Syst. BP Baseline	133 ± 13	135 ± 15	0,575
Syst. BP Peak	175 ± 21	173 ± 27	0,640
%theoretical MHR	95 ± 7	91 ± 9	0,02
Duration seconds	659 ± 159	503 ± 175	0,000
Time recovery HR	254 ± 99	260 ± 151	0,832
Double product	28760 ± 4493	26232 ± 4760	0,015
Angina during ESE	22/33 (66%)	20/58(34%)	0,002

HR – heart rate; BP – blood pressure; MHR – maxymal heart rate.

**Table 3:** Details of echocardiogram M Mode

	Group A	Group B	p
LVEDDi (mm/m^2^)	25,3 ± 2,8	28 ± 2,7	0,000
LVESDi	15,6 ± 2,4	17,4 ± 2,3	0,0002
FS (%)	38,9 ± 5,4	37,5 ± 4,9	0,219
IVSi (mm/m^2^)	5,2 ± 0,9	5,1 ± 0,8	0,62
PWi (mm/m^2^)	4,55 ± 0,7	4,59 ± 0,6	0,75
LVMi g/m^2^	73,9 ± 13,1	80,6 ± 13,9	0,028
LA (mm)	37,1 ± 3,2	37,8 ± 2,7	0,279
RLVWT	0,36 ± 0,068	0,33 ± 0,046	0,01

LVEDDi – left ventricle telediastolic diameter index; LVESDi – left ventricle telesystolic diameter index; FS – fraccional shortening; IVSi – interventricular septum index; PWi – posterior wall index; LVMi – left ventricular mass index; LA – left atrium; RLVWT – relative left ventricular wall thickness

**Table 4:** Details of two-dimensional echocardiogram

	Group A	Group B	p
LVOTi (mm/m^2^)	10,29 ± 0,9	11,4 ± 1	0,000
EF (%)	67,94 ± 5,4	66,90 ± 4,5	0,333
LVDVi ml/m^2^	44,8 ± 10	56 ± 11,6	0,000
D1 (mm)	10,72 ± 3,11	13,75 ± 2,98	0,000

LVOTi – left ventricular outflow tract index; EF – ejection fraction, LVDVi – left ventricular diastolic volume index; D1 – distance D1 measured as explained in figure 1.

**Table 5:** Details of echocardiogram (Doppler)

	Group A	Group B	p
CiLLD ml/m^2^	2086 ± 561	2235 ± 495	0,198
CWmáxLLD cm^s^	130 ± 15,8	120,6 ± 12,5	0,002
CWmáxOrtho cm^s^	117 ± 14	111 ± 12	0,027
CWmáx 3	182 ± 15	158 ± 15	0,000
E cm^s^	85 ± 14	85 ± 16	0,963
A cm^s^	68 ± 19	67 ± 13	0,772
Dec. time sec.	170 ± 34	175 ± 44	0,614
IVRT	85,9 ± 15	88,9 ± 11	0,286
PV	50 ± 12,8	47,9 ± 9,8	0,314

CiLLD – cardiac index in left lateral decubitus before de start of the exam; CWmáxLLD – máximal velocity of flow obtained at apical five chamber view with continuous Doppler oriented through LVOT to the aorta in left lateral decubitus; CwmáxOrtho – máximal velocity of flow obtained at apical five chamber view with continuous Doppler oriented through LVOT to the aorta in orthostatic position; CWmáx3-máximal velocity of flow obtained at apical five chamber view with continuous Doppler oriented through LVOT to the aorta; E- maximal velocity of E wave of mitral flow; A – maximal velocity of E wave of mitral flow; Dec. Time sec.- deceleration time in seconds; IRVT – isovolumic relaxation time; PV propagation of velocity evaluated with M Mode color.

In group A 23 pts (70%) develop SAM (Figure 2, Additional file 3) during exercise, associated with IVG (Figure 3, Additional file 2). No one patient developed segmental wall abnormalities.

### Multivariate Analysis

A logistic regression model was constructed with the following variables: age, sex, effort angina, left ventricular outflow tract index, left ventricular diastolic volume index, relative wall thickness, left ventricular mass index, D1 distance. From the variables included attained statistical significance (p < 0.05) the contribution of effort angina, D1 distance, LVDVi, LVOTi and sex, for appearance of IVG as we can see in Table 6.

**Table 6:** Multivariate analysis

Variable	-2 Log Likelihood	Loss Function (p)
Age	114,1548	,071918
Sex	109,0601	,023868
LVOTi (mm/m^2^)	91,70272	,000031
LVDVi ml/m^2^	81,35754	,001299
RLVWT	78,80142	,109878
LVMi g/m^2^	78,73733	,800141
D1 distance	64,62039	,000172
Effort Angina	52,25502	,000438

LVOTi; LVDVi; RLVWT; LVMi; D1 – as previously defined

## Discussion

Patients with a positive treadmill exercise test, and normal coronary angiography have long been recognised as an important problem in clinical practice [8-10]. These early studies identified many of the characteristics of what was subsequently characterized as syndrome X [10]. The same denomination was also applied to a syndrome, characterized by insulin resistance, hyperinsulinemia, and diabetes, that is associated with dyslipidemia, hypertension, and abdominal obesity. Hence a more specific terminology comes in use: angina with normal coronary arteriography [11]. Patients with this entity, predominantly women [12], complain of pain that is frequently atypical. It may be precipitated by exertion, although the threshold for precipitating pain is highly variable [13]. Its duration may be uncharacteristically long, and it may be unusually severe and is rarely associated with symptoms such as diaphoresis. Perfusion abnormalities have been observed commonly in patients with chest pain and normal coronary arteriograms, but no consistent correlation could be made among the extent of the defect, the positivity of the exercise test, and exercise tolerance [14]. Thus in many of this patients there is evidence of perfusion abnormalities that are attributed to abnormalities in the microvasculature [15]. However stress echocardiography allways failled to demonstrate segmental wall abnormalities even showing hyperdinamic ventricles[16].

The results of our study, in which 33 (36%) of 91 patients with normal coronary angiogram and positive treadmill exercise test developed intraventricular gradient, suggest that ST-segment depression may be related with the development of IVG during exercise which is possibly involved in the genesis of electrocardiographic changes. The possible association between cardíac X syndrome and the development of IVG during exercise was described before [17,18] however some of the patients from these studies have arterial hypertension, and left ventricular hypertrophy that by definition of X Syndrome [19] we have excluded and that more frequently developed IVG [3].

The appearance of IVG in our study was associated with morphological determinants like reduced LVOTi, reduced left ventricular diastolic volume, a reduced distance D1, and increased relative left ventricular wall thickness. All these finding translate a proportional small heart that the multivariate model confirms.

The reduced D1 in Group A means an anterior "displacement" of the postero internal papillary muscle that may be involved in the development of IVG and SAM of the mitral valve [20,21] as described by other authors.

We can admit that this phenomenon is eventually caused by the subtle changes in left ventricle geometric shape and dimensions with more anterior papillary muscles implantation [20,21], that during exercise, induce and submit the cordae and mitral valve to an abnormal systolic anterior motion and to papillary muscle ischemia. The obstruction to the outflow in left ventricle with the increase in the intraventricular pressure that it causes may contribute, to left ventricular strain and ST-depression in this patients.

The development of intraventricular gradient during exercise may possibly explain the ST changes in a subgroup of patients who have treadmill positive test and normal coronary arteries.

The patients with IVG during exercise had more angina during exercise and were predominantly male, and this may explain why these patients were submitted to coronary angiography much early, after the beginning of the symptons, than patients in Group B. From the all study group 42 patients (46%) reproduced symptoms during ESE, however this fact occured more frequently (22 pts from 33 in group A vs 20 from 58 pts in group B – p = 0.002) in group A, favouring the potencial participation of intraventricular gradient in the occurrence of symptoms.

In our study population, we found a great number of patients that develop SAM of the mitral valve in association with IVG contrarily to other authors [17,18]. We think that we detect SAM in a greater number of patients because we do echo during all the exercise in treadmill (Additional file 2 and 4) [7]. The magnitude of the IVG that we have detected in our patients is also greater for the same motive (Figure 3).

Four of the 33 patients that developed intraventricular gradient are athletes [22] and we should probably study this phenomenon in this specific population and, if this occurs, also investigate the possible prognostic implications for this event in this particular population [23].

The results of ESE have probably influenced the treatment of the patients once at the end of follow-up a greater percentage of patients are treated with β blockers [24,25] in group A than in group B (Table 1).

The principal limitations of this study are: 1) no one patient has done a test for provocation of coronary spasm at cath. laboratory even no patient included in the study had segmental wall abnormalities with exercise 2) The presence or absence of ischemia was only evaluated by ESE without use of scintigraphic studies. 3) We excluded all patients with left ventricular hypertrophy and uncontroled arterial hypertension that constitutes a great number of patient in the real world of clinical practice and that should be studied in the future with the same protocol.

## Conclusion

We can conclude that a relevant number of patients with cardiac X syndrome develop significant intraventricular gradient during exercise and also that morphological variables are involved in is pathophysiology. The authors believe that this phenomenon may constitute a new entity that joins to the heterogeneous group of patients with angina, ST-depression during treadmill exercise test and normal coronary arteriography.

As as consequence of our results, exercise stress echocardiography should be part of new diagnostic algorithm whenever we suspect that our patients with angina may have cardiac X syndrome.

## Supplementary Material

Additional file 1 Echocardiographic images obtained during exercise. Apical four and five chamber view obtained in apical window during exercise containing two dimensional and Doppler data. Click here for file

Additional file 2 Images obtained during exercise test in the first patient with IVG. Images obtained during the exam that we repeated, after informed consent was obtained, in the first patient included in the study. IVG is easily observed during exercise echo. Click here for file

Additional file 3 Images obtained during exercise test in the first patient with IVG and SAM. Images obtained during the exam that we repeated, after informed consent was obtained, in the first patient included in the study. SAM of mitral valve is easily observed during exercise echo. Click here for file

Additional file 4 Images obtained during exercise test. Images obtained during exercise test showing the position of operator with the cubital border of the right hand attatched to the patient chest wall. Click here for file
